# Supplementary material for: RNA and DNA G-quadruplexes bind to human dicer and inhibit its activity
Source: Cell Mol Life Sci. 2021 Mar 17;78(7):3709–24. doi: 10.1007/s00018-021-03795-w (PMC8038994; doi:10.1007/s00018-021-03795-w)
Supplement: Supplementary file 3 — Supplementary file3 (PDF 1102 KB) [file 18_2021_3795_MOESM3_ESM.pdf]

Supplementary Information for

RNA and DNA G-quadruplexes bind to human Dicer and inhibit its activity

Natalia Koralewska, Agnieszka Szczepanska, Kinga Ciechanowska, Marta Wojnicka, Maria Pokornowska, Marek C. Milewski, Dorota Gudanis, Daniel Baranowski, Chandran Nithin, Janusz M. Bujnicki, Zofia Gdaniec, Marek Figlerowicz and Anna Kurzynska-Kokorniak

Anna Kurzynska-Kokorniak
Email: akurzyns@man.poznan.pl

This PDF file includes:

Supplementary Methods
Figures S1 to S7
Table S1
Table S2
Legend for Supplementary File S1
Legend for Dataset S1
Supplementary References

Other supplementary materials for this manuscript include the following:

Supplementary File S1
Dataset S1

Supplementary Methods

^{32}P labeling of oligonucleotides

The 5′-end labeling was performed using 10 pmol of RNA/DNA, 1 μL γ-^{32}P-ATP (3000 Ci/mmol, Hartman Analytic GmbH) and 10 U T4 polynucleotide kinase (Thermo Fisher Scientific) in a final volume of 10 μL. After 10 min of the incubation at 37°C, the reaction was halted by adding 1 μL 0.5 M EDTA, pH 8.0. The radiolabeled oligonucleotides were PAGE-purified in 8% denaturing polyacrylamide gels and resuspended in water to a final concentration of approximately 10,000 cpm/μL.

RNA-RNA binding assay

RNA-RNA complex formation was analyzed using EMSA. The specific 5′-^{32}P-labeled pre-miRNA (10,000 cpm, approximately 5 nM) was mixed with the respective guanine-rich oligomer (100 pmol), and incubated in binding buffer (20 mM Tris-HCl, pH 7.5, 100 mM KCl) in a final volume of 10 μL for 30 min at 37°C. The samples were separated in 12% non-denaturing polyacrylamide gels supplemented with 5% glycerol at room temperature for approximately 6 h at 7 V/cm in 1×TBE.

hDicer production

Human Dicer was prepared as described previously [1]. In brief, protein with a His-tag at the C terminus was expressed in Baculovirus Expression System (Life Technologies) and purified by Ni^{2+} affinity chromatography (Ni-NTA Agarose, Qiagen) followed by ion-exchange chromatography (HiTrap Q HP, GE Healthcare). The protein preparation was assessed by SDS-PAGE and the enzyme activity was assayed as reported previously [1]. Non-reducing conditions were applied for both protein purification and storage.

Docking and molecular modeling

Docking of RNA and the protein was performed following a meta-approach using different docking methods to generate the docking poses, followed by the rescoring and selection of best poses [2]. The hDicer (PDB entry 4NGF) and G-quadruplex (PDB entry 2M18) were docked using the following methods: 3dRPC/RPDOCK [3], ClusPro [4], HADDOCK [5], HDOCK [6], Hex [7], PatchDock [8] and ZDOCK [9]. Additionally, the protein-RNA complex structures were generated with an in-house method SimRNP developed by the Bujnicki group (Michał Boniecki and. J.M.B., unpublished) as an extension of SimRNA [10].

From each of the methods, 100 top-scored docking poses (according to the internal scoring system of each method) were selected, resulting in a total of 1200 decoys. These selected top poses were re-scored using four different scoring functions: QUASI-RNP [11], ProSPR (S. Mukherjee, personal communication), 3dRPC/RPRANK [12], and ITScorePR [13]. From each of these scoring trials, 100 top-scored poses were collected. These poses were superposed onto the coordinates of the cryo-EM structure of hDicer in complex with a pre-miRNA substrate (PDB ID: 5ZAL) [14] and inspected for clash with the pre-miRNA. The poses which clash with the pre-miRNA binding were selected.

Molecular dynamics simulations were performed with the selected poses using the Amber 18 package [15]. The missing residues in the hDicer protein structure were built using the MODELLER [16] module in UCSF Chimera [17]. The input structure for the simulation was prepared using tleap in a truncated octahedral box of 10 Å allowance using the TIP3P water model [18]. The simulations were performed using the combination of the Amber ff14sb force field [19] for proteins and the χOL3 force field [20] for RNA. The structure was energy-minimized for 10,000 cycles with restraints, followed by 10,000 cycles without restraints. The minimized structures were subjected to heating, density equilibration and short runs of equilibration. The heating was done from 100 K to 300 K for 500 ps with restraints on the entire structure and the density equilibration was performed for 500 ps, also with restraints on the entire structure. The equilibration of the structures was run for four short rounds. The first three rounds of equilibration were run for 200 ps each with the main chain atoms constrained. The final round of

equilibration was performed for 2 ns. The production run was run for 100 ns. The minimization was performed using Sander, and the subsequent steps were performed using the CUDA version of PMEMD available in the Amber package [21-23]. The simulation trajectory (Supplementary Figure S7) was clustered and the representative frame is provided as PDB file in the Supplementary Data (Supplementary File S1).

Docking of G4T4G4 DNA (PDB ID: 1JPQ) and the hDicer protein (PDB ID: 4NGF) was performed using the following docking methods: ClusPro [4], HADDOCK [5], HDOCK [6], Hex [7], PatchDock [8] and ZDOCK [9]. While HADDOCK have modules to dock protein and DNA explicitly, ClusPro allows only use of DNA as a receptor molecule. For the methods HDOCK, Hex, PatchDock and ZDOCK the docking was performed both with protein as receptor and DNA as ligand, and vice-versa. From each of these docking trials, 100 top scored docking poses (according to the internal scoring system of each method) were selected resulting in a total of 1000 decoys. These poses were superposed on to the cryo-EM structure of hDicer in complex with a pre-miRNA substrate (PDB ID: 5ZAL) [14] and inspected for clash with the pre-miRNA substrate. The poses that clash with the pre-miRNA binding were selected.

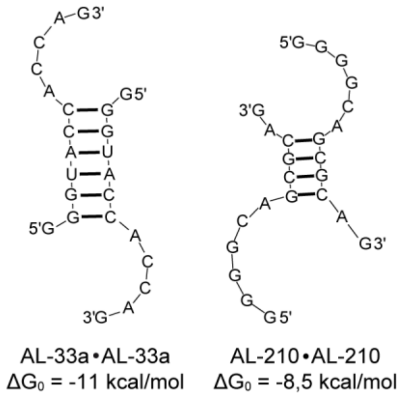

Fig. S1 AL-33a and AL-210 display potential for homodimerization. Schematic representation of the secondary structure predicted for AL-33 (*left*) and AL-210 (*right*).

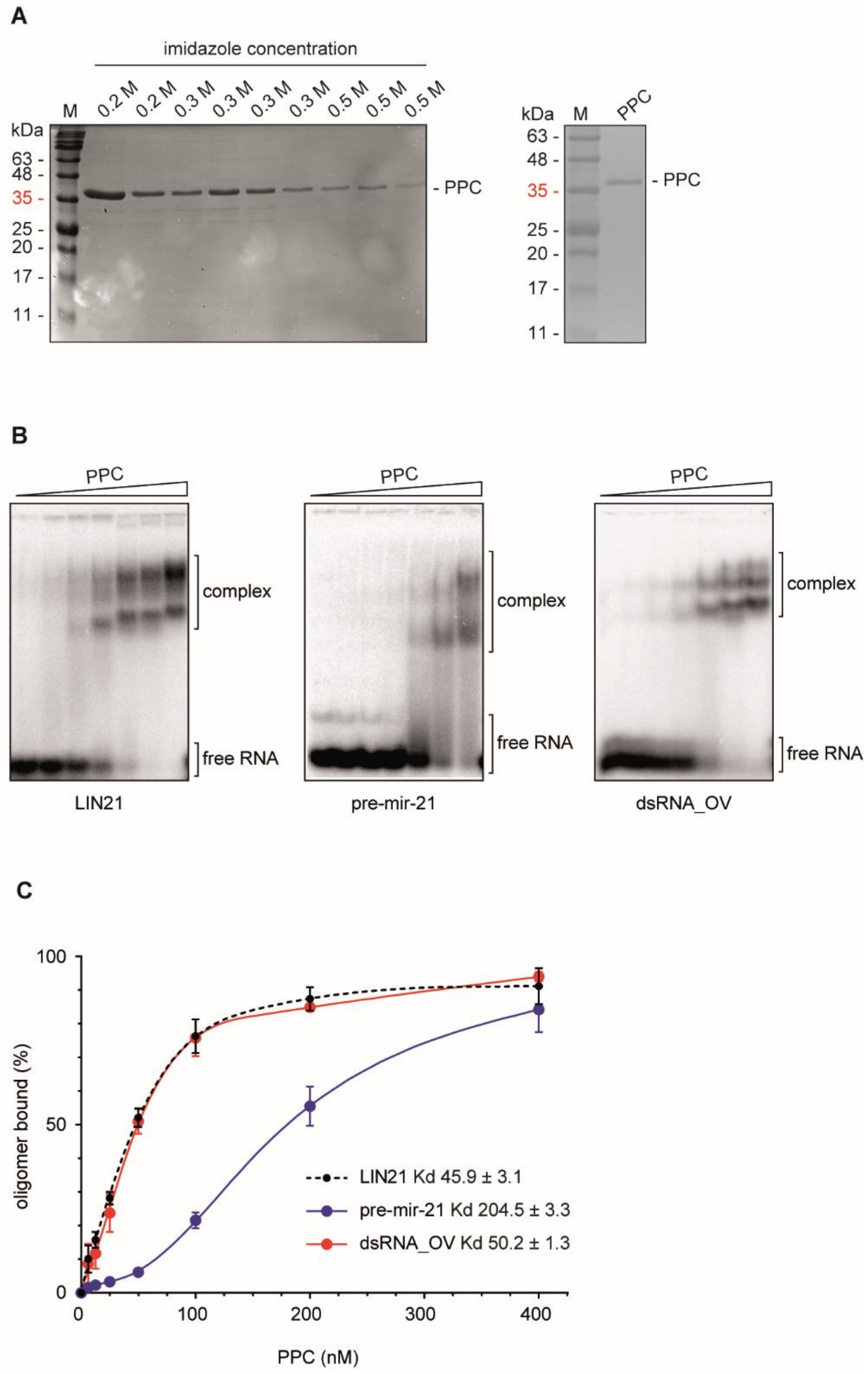

Fig. S2 Biochemical characteristics of hDicer PPC cassette. (A) SDS-PAGE analysis of the fractions collected after purification of PPC using Ni^{2+}- affinity chromatography (*left*) and the final PPC preparation used in the further assays (*right*). The analyzed protein fractions were washed out with buffers containing different imidazole concentration as indicated. Proteins were detected by Coomassie brilliant blue staining. M – protein size marker (11-245 kDa, EURx). (**B**) EMSAs with PPC and 5′-^{32}P-labeled ssRNA: 21-nt LIN21, 58-nt pre-mir-21 or dsRNA: 19-bp dsRNA_OV with 2-nt 3′-overhangs. Triangles represent increasing amount of PPC (6.25, 12.5, 25, 50, 100, 200, 400 nM). Free RNA and the RNA in complex with PPC are indicated. (**C**) The Kd values of RNA-PPC complexes were determined from the binding isotherms by curve fitting using nonlinear regression. Error bars represent SD from three separate experiments.

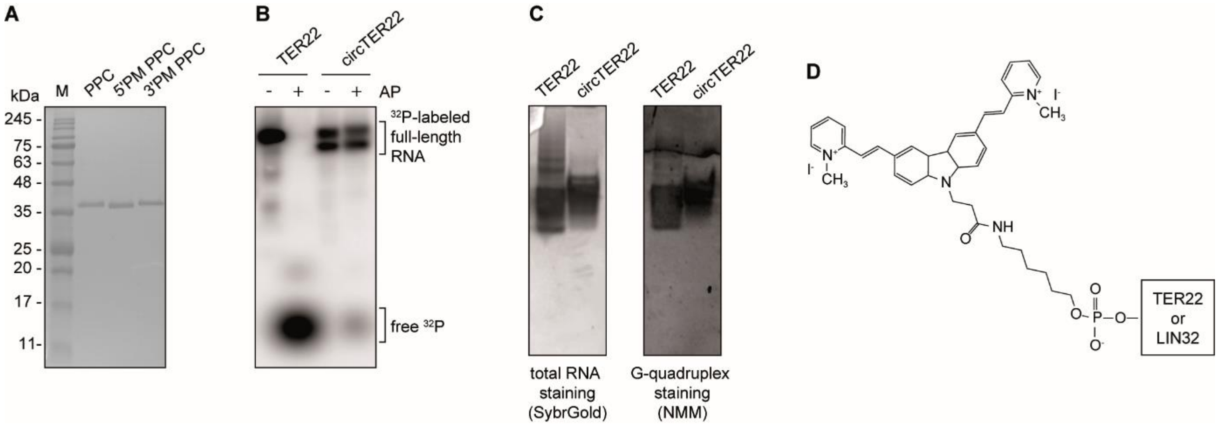

Fig. S3 Characterization of hDicer PPC cassette preparations and G-quadruplexes used for binding assays. (A) SDS-PAGE analysis of wild-type PPC and PPC 3′- and 5′-pocket mutants (PM). M – protein size marker (11-245 kDa, EURx). **(B)** Verification of the efficiency of TER22 circularization. Shrimp alkaline phosphatase (AP) dephosphorylates 5′-^{32}P-labeled TER22 with free ends but not after successful 5′-3′ end ligation. **(C)** Native PAGE analysis of linear or circularized TER22. Gels were treated with nucleic acid stain SYBR Gold (*left*) or G-quadruplex-specific dye, NMM (*right*). **(D)** Schematic representation of the structure of *o*-BMVC-C3-labeled oligomers.

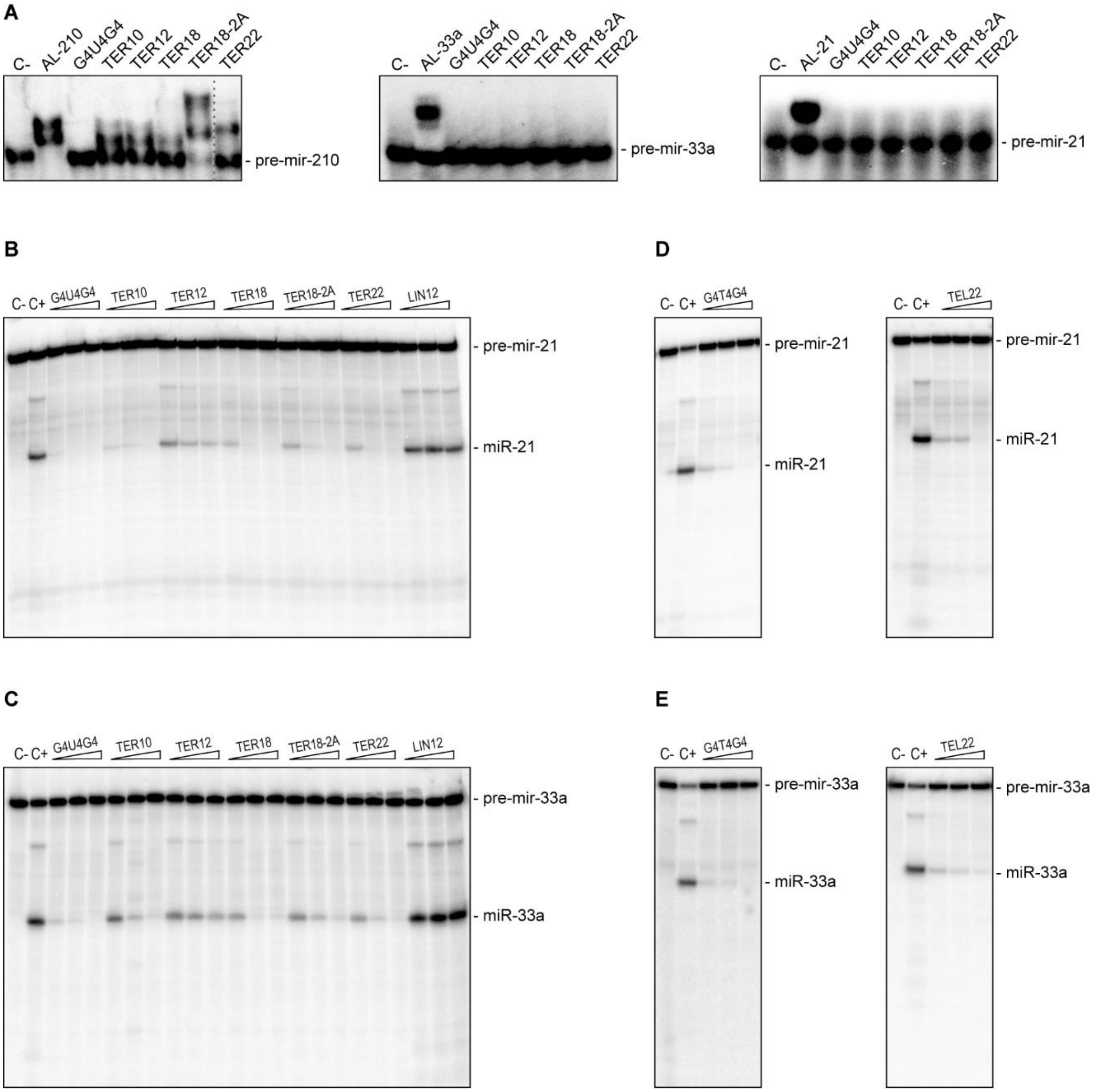

Fig. S4 Characterization of the effect exerted by G-quadruplexes on pre-miRNA cleavage by Dicer. **(A)** Native PAGE analysis of 5′-^{32}P-labeled (10,000 cpm, approximately 5 nM) pre-mir-210, pre-mir-33a, pre-mir-21 incubated with a complementary 12-nt long RNA (AL-210, AL-33 or Al-21, respectively) and a set of RNA G-quadruplexes (10 μM). The position of a band corresponding to free pre-miRNA is indicated. Pre-mir-33a and pre-mir-21, as pre-miRNAs non-interacting with tested G-rich oligomers, were used as substrates in Dicer cleavage inhibition assays. *C-* a sample with pre-miRNA only. **(B, C)** Representative results of PAGE analysis used to quantify the effect of RNA G-quadruplexes (G4U4G4, TER10, TER12, TER18, TER18-2A, TER22) on the cleavage of pre-mir-21 (B) or pre-mir-33a (C) by hDicer. *C-* a sample with no protein, nor inhibitor added, *C+* a sample with hDicer, without inhibitor. Triangles represent an increasing amount (0.1, 0.5, 2 μM) of the indicated oligomer. LIN12 – a control 12-mer not adopting a G-quadruplex structure. The reproducible results were obtained using at least two batches of recombinant hDicer. **(D, E)** Representative results of PAGE analysis used to quantify the effect of DNA G-quadruplexes (G4T4G4, TEL22) on the cleavage of pre-mir-21 (D) or pre-mir-33a (E) by

hDicer. *C-* a sample with no protein, nor inhibitor added, *C+* a sample with hDicer, without inhibitor. Reactions were carried out for 30 min at 37°C. Triangles represent increasing amount of the indicated oligomer (0.1, 0.5, 2 μM). The reproducible results were obtained using at least two batches of recombinant hDicer.

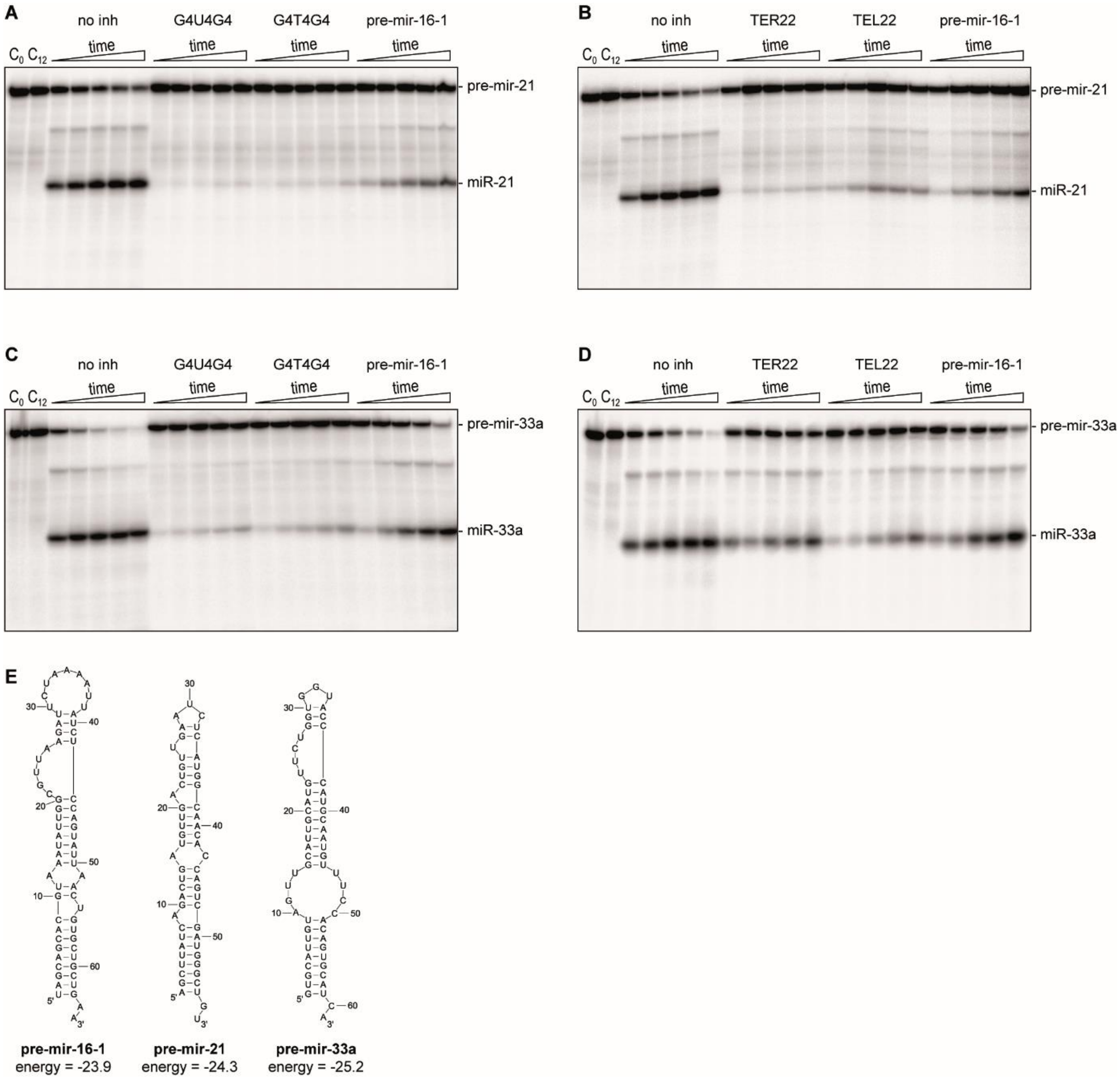

Fig. S5 The time course for inhibition of pre-miRNA cleavage by Dicer mediated by G-quadruplexes under low-turnover conditions. (A-D) Representative PAGE results used for quantitative analysis of the time course of hDicer (10 nM) inhibition by RNA and DNA G-quadruplexes (500 nM) in reactions with 5′-^{32}P-labeled pre-miRNA (10,000 cpm, approximately 5 nM): pre-mir-21 (A, B) or pre-mir-33a (C, D). Reactions were carried out at 37°C for: 30 min, 1.5 h, 3 h, 6 h, 12 h (increasing incubation time is indicated by a triangle). C_0, C_{12} controls with no oligomer, nor hDicer added, stopped immediately after reaction assembly or after 12 h of incubation, respectively. The reproducible results were obtained using at least two batches of recombinant hDicer. **(E)** The lowest free energy structures predicted by RNAstructure Fold algorithm for pre-miRNAs used in the assay. The free energy values are expressed in kcal/mol.

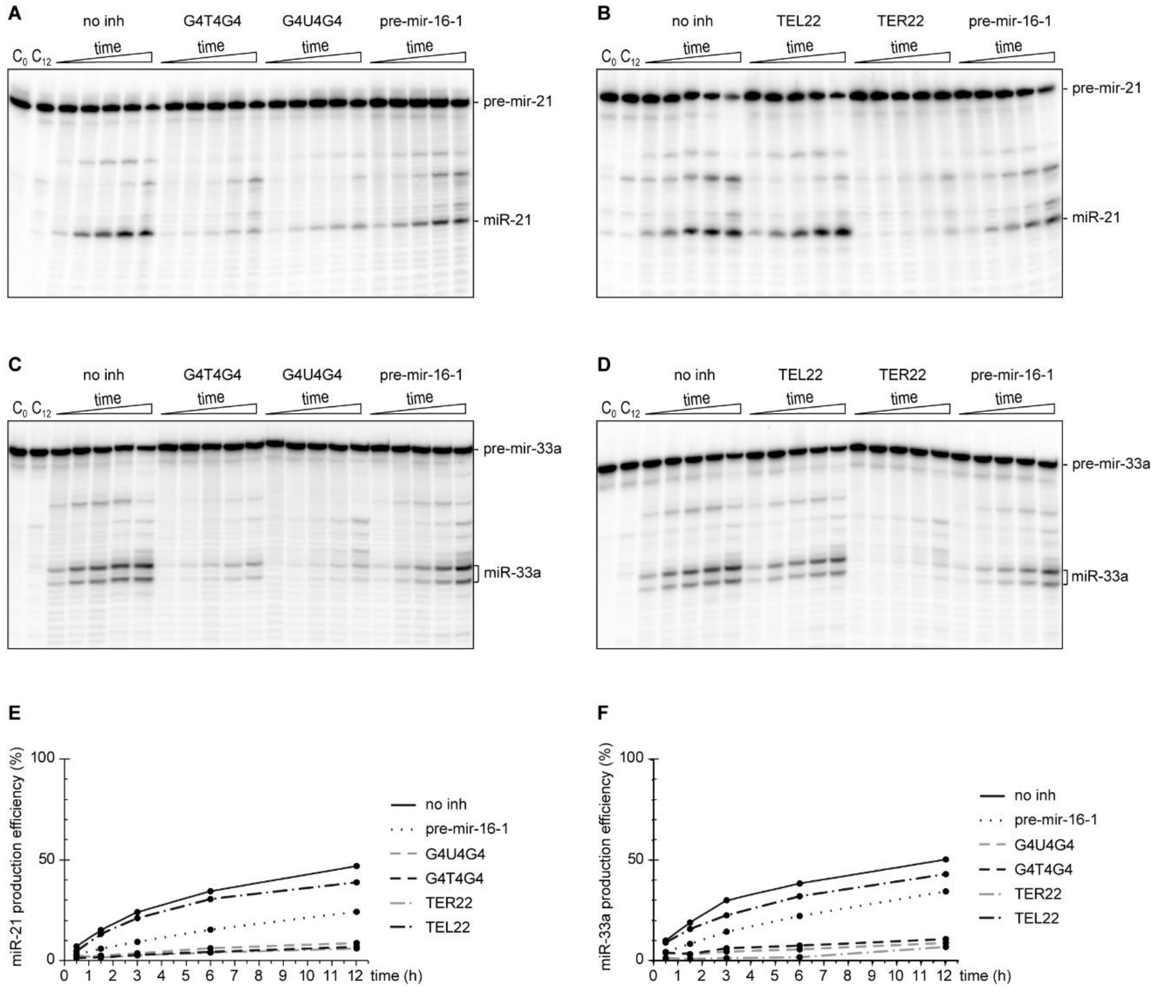

Fig. S6 The time course for inhibition of pre-miRNA cleavage by Dicer mediated by G-quadruplexes under high-turnover conditions. (A-D) PAGE results used for quantitative analysis of the time course of hDicer (0.5 nM) inhibition by RNA and DNA G-quadruplexes (50 nM) in reactions with 5′-^{32}P-labeled pre-miRNA (100,000 cpm, approximately 25 nM): pre-mir-21 (A, B) or pre-mir-33a (C, D). Reactions were carried out at 37°C for: 30 min, 1.5 h, 3 h, 6 h, 12 h (increasing incubation time is indicated by a triangle). C_0, C_{12} controls with no oligomer, nor hDicer added, stopped immediately after reaction assembly or after 12 h of incubation, respectively. **(E-F)** Quantitative analysis of the time course of hDicer inhibition by G-quadruplexes in reactions with pre-mir-21 (E) or pre-mir-33a (F). Values are the average from two independent experiments.

A

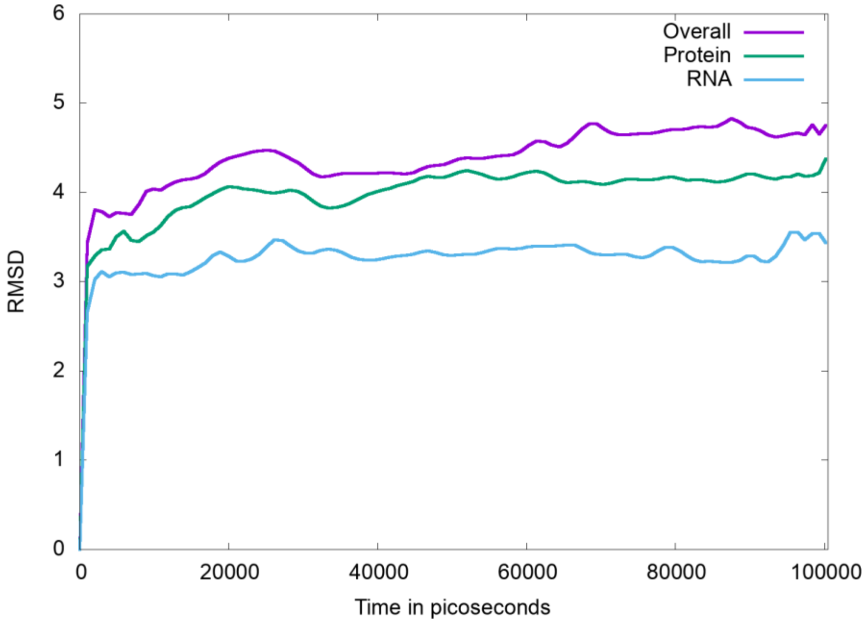

B

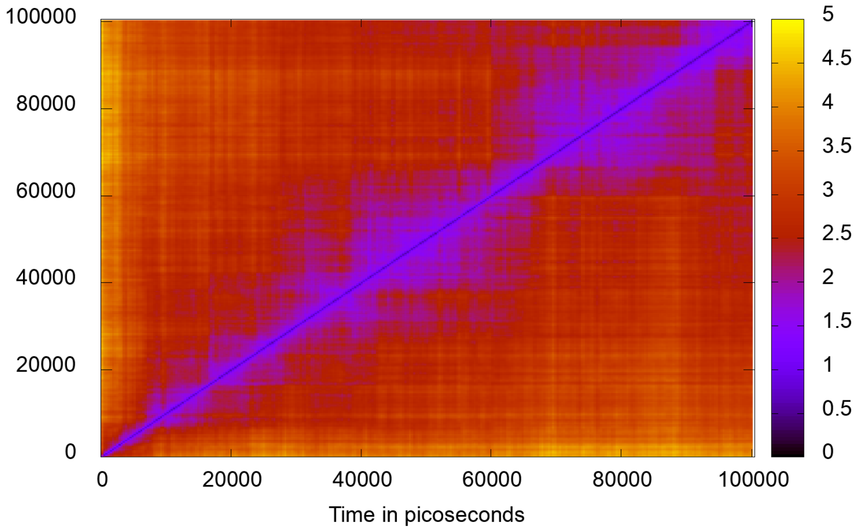

Fig. S7 Molecular dynamics simulations of hDicer and G-quadruplex complex. **(A)** The root mean square deviation (RMSD) of the frames with respect to the starting frame of the simulations remains stable throughout the simulations. **(B)** The heat map of pairwise RMSD of different frames during the MD simulations; points are colored based on the RMSD.

Table S1. Characteristics of RNA and DNA quadruplexes used in the study.

**Name**	**Sequence**	**PDB ID**	**Type**	**Topology**	**Reference**
TER10	r(GGGUUAGGGU)	2M18	dimer of bimolecular quadruplexes	parallel	[24,25]
TER12	r[(UAGGGU)$_2$]	2KBP	bimolecular	parallel	[25]
TER18	r(GGGUUGCGGAGGGUGGGC)	-	monomolecular or dimer of monomolecular quadruplexes	parallel	[26]
TER18-2A	r(AAGGGUUGCGGAGGGUGGGC)	-	monomolecular	parallel	[26]
TER22	r[AGGG(UUAGGG)$_3$]	-	monomolecular	parallel	[27]
G4U4G4	r(GGGGUUUUGGGG)	-	bi- or tetramolecular	parallel	[28]
QU14	r(GGAGGUUUUGGAGG)	1MY9	dimer of monomolecular quadruplexes	parallel	[29]
TEL22	d[AGGG(TTAGGG)$_3$]	-	monomolecular	mixture of parallel/antiparallel	[30]
G4T4G4	d(GGGGTTTTGGGG)	1JPQ	bimolecular	antiparallel	[31,32]

Table S2. Sequences of all oligonucleotides used in the binding and cleavage assays.

Name	Sequence (5′ → 3′)
AL-16-1	GAAUCUUAACGC
AL-21	AUGAGAUUCAAC
AL-33a	GGGUACCACCAG
AL-210	GGGGCAGCGCAG
LIN21*	UCGAAGUAUUCCGCGUACGUG
LIN32	GUGCAUUGUAGUUGCAUUGCAUGUUCUGGUCA
dsRNA_OV	CGUACGCGGAAUACUUCGAAA
TER10	GGGUUAGGGU
TER12	UAGGGUUAGGGU
TER18	GGGUUGCGGAGGGUGGGC
TER18-2A	AAGGGUUGCGGAGGGUGGGC
TER22	AGGGUUAGGGUUAGGGUUAGGG
TEL22	AGGGTTAGGGTTAGGGTTAGGG
G4U4G4	GGGGUUUUGGGG
G4T4G4	GGGGTTTTGGGG
QU14	GGAGGUUUUGGAGG
pre-mir-16-1	UAGCAGCACGUAAAUAUUGGCGUUAAGAUUCUAAAAUUAUCUCCAGUAUUAACUGUGC UGCUGAA
pre-mir-21	AGCUUAUCAGACUGAUGUUGACUGUUGAAUCUCAUGGCAACACCAGUCGAUGGGCUGU
pre-mir-33a	GUGCAUUGUAGUUGCAUUGCAUGUUCUGGUGGUACCCAUGCAAUGUUUCCACAGUGCAU CA
pre-mir-210	GCCCCUGCCCACCGCACACUGCGCUGCCCCAGACCCACUGUGCGUGUGACAGCGGCUG

** LIN21 also serves as a complementary strand to form dsRNA with dsRNA_OV*

Supplementary File S1 (separate file). A representative frame from the molecular dynamics simulation of hDicer and G-quadruplex complex.

Dataset S1 (separate file). Guanine-rich sequences identified in the transcriptome-wide map of hDicer targets.

Supplementary References

1. Pokornowska M, Milewski MC, Ciechanowska K, Szczepanska A, Wojnicka M, Radogostowicz Z, Figlerowicz M, Kurzynska-Kokorniak A (2019) The RNA-RNA base pairing potential of human Dicer and Ago2 proteins. Cell Mol Life Sci. doi:10.1007/s00018-019-03344-6
2. Nithin C, Ghosh P, Bujnicki JM (2018) Bioinformatics Tools and Benchmarks for Computational Docking and 3D Structure Prediction of RNA-Protein Complexes. 9 (9):432. doi: 10.3390/genes9090432
3. Huang Y, Liu S, Guo D, Li L, Xiao Y (2013) A novel protocol for three-dimensional structure prediction of RNA-protein complexes. Scientific Reports 3:1887. doi:10.1038/srep01887
4. Comeau SR, Gatchell DW, Vajda S, Camacho CJ (2004) ClusPro: a fully automated algorithm for protein–protein docking. Nucleic Acids Research 32 (suppl_2):W96-W99. doi:10.1093/nar/gkh354
5. van Zundert GCP, Rodrigues JPGLM, Trellet M, Schmitz C, Kastritis PL, Karaca E, Melquiond ASJ, van Dijk M, de Vries SJ, Bonvin AMJJ (2016) The HADDOCK2.2 Web Server: User-Friendly Integrative Modeling of Biomolecular Complexes. Journal of Molecular Biology 428 (4):720-725. doi:https://doi.org/10.1016/j.jmb.2015.09.014
6. Yan Y, Zhang D, Zhou P, Li B, Huang S-Y (2017) HDOCK: a web server for protein–protein and protein–DNA/RNA docking based on a hybrid strategy. Nucleic Acids Research 45 (W1):W365-W373. doi:10.1093/nar/gkx407
7. Macindoe G, Mavridis L, Venkatraman V, Devignes M-D, Ritchie DW (2010) HexServer: an FFT-based protein docking server powered by graphics processors. Nucleic Acids Research 38 (suppl_2):W445-W449. doi:10.1093/nar/gkq311
8. Schneidman-Duhovny D, Inbar Y, Nussinov R, Wolfson HJ (2005) PatchDock and SymmDock: servers for rigid and symmetric docking. Nucleic Acids Research 33 (suppl_2):W363-W367. doi:10.1093/nar/gki481
9. Pierce BG, Wiehe K, Hwang H, Kim B-H, Vreven T, Weng Z (2014) ZDOCK server: interactive docking prediction of protein–protein complexes and symmetric multimers. Bioinformatics 30 (12):1771-1773. doi:10.1093/bioinformatics/btu097
10. Boniecki MJ, Lach G, Dawson WK, Tomala K, Lukasz P, Soltysinski T, Rother KM, Bujnicki JM (2015) SimRNA: a coarse-grained method for RNA folding simulations and 3D structure prediction. Nucleic Acids Research 44 (7):e63-e63. doi:10.1093/nar/gkv1479
11. Tuszynska I, Bujnicki JMJBB (2011) DARS-RNP and QUASI-RNP: New statistical potentials for protein-RNA docking. 12 (1):348. doi:10.1186/1471-2105-12-348
12. Huang Y, Li H, Xiao YJBR (2016) Using 3dRPC for RNA–protein complex structure prediction. 2 (5):95-99. doi:10.1007/s41048-017-0034-y
13. Huang S-Y, Zou X (2014) A knowledge-based scoring function for protein-RNA interactions derived from a statistical mechanics-based iterative method. Nucleic acids research 42 (7):e55-e55. doi:10.1093/nar/gku077
14. Liu Z, Wang J, Cheng H, Ke X, Sun L, Zhang QC, Wang H-W (2018) Cryo-EM Structure of Human Dicer and Its Complexes with a Pre-miRNA Substrate. Cell 173 (5):1191-1203.e1112. doi:https://doi.org/10.1016/j.cell.2018.03.080
15. Salomon-Ferrer R, Case DA, Walker RC (2013) An overview of the Amber biomolecular simulation package. 3 (2):198-210. doi:10.1002/wcms.1121
16. Eswar N, Webb B, Marti-Renom MA, Madhusudhan MS, Eramian D, Shen MY, Pieper U, Sali A (2006) Comparative protein structure modeling using Modeller. Current protocols in bioinformatics Chapter 5:Unit-5.6. doi:10.1002/0471250953.bi0506s15
17. Pettersen EF, Goddard TD, Huang CC, Couch GS, Greenblatt DM, Meng EC, Ferrin TE (2004) UCSF Chimera--a visualization system for exploratory research and analysis. Journal of computational chemistry 25 (13):1605-1612. doi:10.1002/jcc.20084

18. Sun YX, Kollman PA (1995) Hydrophobic Solvation of Methane and Nonbond Parameters of the Tip3p Water Model. Journal of computational chemistry 16 (9):1164-1169. doi:DOI 10.1002/jcc.540160910
19. Maier JA, Martinez C, Kasavajhala K, Wickstrom L, Hauser KE, Simmerling C (2015) ff14SB: Improving the Accuracy of Protein Side Chain and Backbone Parameters from ff99SB. J Chem Theory Comput 11 (8):3696-3713. doi:10.1021/acs.jctc.5b00255
20. Banáš P, Hollas D, Zgarbová M, Jurečka P, Orozco M, Cheatham TE, Šponer J, Otyepka M (2010) Performance of Molecular Mechanics Force Fields for RNA Simulations: Stability of UUCG and GNRA Hairpins. Journal of Chemical Theory and Computation 6 (12):3836-3849. doi:10.1021/ct100481h
21. Le Grand S, Götz AW, Walker RC (2013) SPFP: Speed without compromise—A mixed precision model for GPU accelerated molecular dynamics simulations. Computer Physics Communications 184 (2):374-380. doi:10.1016/j.cpc.2012.09.022
22. Gotz AW, Williamson MJ, Xu D, Poole D, Le Grand S, Walker RC (2012) Routine Microsecond Molecular Dynamics Simulations with AMBER on GPUs. 1. Generalized Born. J Chem Theory Comput 8 (5):1542-1555. doi:10.1021/ct200909j
23. Salomon-Ferrer R, Gotz AW, Poole D, Le Grand S, Walker RC (2013) Routine Microsecond Molecular Dynamics Simulations with AMBER on GPUs. 2. Explicit Solvent Particle Mesh Ewald. J Chem Theory Comput 9 (9):3878-3888. doi:10.1021/ct400314y
24. Martadinata H, Phan AT (2013) Structure of human telomeric RNA (TERRA): stacking of two G-quadruplex blocks in K(+) solution. Biochemistry 52 (13):2176-2183. doi:10.1021/bi301606u
25. Martadinata H, Phan AT (2009) Structure of propeller-type parallel-stranded RNA G-quadruplexes, formed by human telomeric RNA sequences in K+ solution. J Am Chem Soc 131 (7):2570-2578. doi:10.1021/ja806592z
26. Martadinata H, Phan AT (2014) Formation of a stacked dimeric G-quadruplex containing bulges by the 5'-terminal region of human telomerase RNA (hTERC). Biochemistry 53 (10):1595-1600. doi:10.1021/bi4015727
27. Zhang DH, Fujimoto T, Saxena S, Yu HQ, Miyoshi D, Sugimoto N (2010) Monomorphic RNA G-quadruplex and polymorphic DNA G-quadruplex structures responding to cellular environmental factors. Biochemistry 49 (21):4554-4563. doi:10.1021/bi1002822
28. Vondruskova J, Kypr J, Kejnovska I, Fialova M, Vorlickova M (2008) Role of loops in the guanine quadruplex formation by DNA/RNA hybrid analogs of G4T4G4. Int J Biol Macromol 43 (5):463-467. doi:10.1016/j.ijbiomac.2008.08.013
29. Liu H, Kugimiya A, Matsugami A, Katahira M, Uesugi S (2002) Quadruplex structures of RNA 14-mer, r(GGAGGUUUUGGAGG) and DNA 14-mer, d(GGAGGTTTTGGAGG). Nucleic Acids Res Suppl (2):177-178. doi: 10.1093/nass/2.1.177
30. Qi J, Shafer RH (2007) Human telomere quadruplex: refolding and selection of individual conformers via RNA/DNA chimeric editing. Biochemistry 46 (25):7599-7606. doi:10.1021/bi602392u
31. Smith FW, Feigon J (1993) Strand orientation in the DNA quadruplex formed from the Oxytricha telomere repeat oligonucleotide d(G4T4G4) in solution. Biochemistry 32 (33):8682-8692
32. Haider S, Parkinson GN, Neidle S (2002) Crystal structure of the potassium form of an Oxytricha nova G-quadruplex. J Mol Biol 320 (2):189-200. doi:10.1016/S0022-2836(02)00428-X
